# Towards Renewed Health Economic Simulation of Type 2 Diabetes: Risk Equations for First and Second Cardiovascular Events from Swedish Register Data

**DOI:** 10.1371/journal.pone.0062650

**Published:** 2013-05-09

**Authors:** Aliasghar Ahmad Kiadaliri, Ulf-G. Gerdtham, Peter Nilsson, Björn Eliasson, Soffia Gudbjörnsdottir, Katarina Steen Carlsson

**Affiliations:** 1 Division of Health Economics, Department of Clinical Sciences, Malmö University Hospital, Lund University, Malmö, Sweden; 2 Health Economics & Management, Institute of Economic Research, Lund University, Lund, Sweden; 3 Department of Health Management and Economics, School of Public Health, Tehran University of Medical Sciences, Tehran, Iran; 4 Department of Economics, Lund University, Lund, Sweden; 5 Department of Clinical Sciences, Malmö University Hospital, Lund University, Malmö, Sweden; 6 Department of Medicine, Sahlgrenska University Hospital, University of Gothenburg, Gothenburg, Sweden; Medical University Innsbruck, Austria

## Abstract

**Objective:**

Predicting the risk of future events is an essential part of health economic simulation models. In pursuit of this goal, the current study aims to predict the risk of developing first and second acute myocardial infarction, heart failure, non-acute ischaemic heart disease, and stroke after diagnosis in patients with type 2 diabetes, using data from the Swedish National Diabetes Register.

**Material and Methods:**

Register data on 29,034 patients with type 2 diabetes were analysed over five years of follow up (baseline 2003). To develop and validate the risk equations, the sample was randomly divided into training (75%) and test (25%) subsamples. The Weibull proportional hazard model was used to estimate the coefficients of the risk equations, and these were validated in both the training and the test samples.

**Results:**

In total, 4,547 first and 2,418 second events were observed during the five years of follow up. Experiencing a first event substantially elevated the risk of subsequent events. There were heterogeneities in the effects of covariates within as well as between events; for example, while for females the hazard ratio of having a first acute myocardial infarction was 0.79 (0.70–0.90), the hazard ratio of a second was 1.21 (0.98–1.48). The hazards of second events decreased as the time since first events elapsed. The equations showed adequate calibration and discrimination (C statistics range: 0.70–0.84 in test samples).

**Conclusion:**

The accuracy of health economic simulation models of type 2 diabetes can be improved by ensuring that they account for the heterogeneous effects of covariates on the risk of first and second cardiovascular events. Thus it is important to extend such models by including risk equations for second cardiovascular events.

## Introduction

Health economic simulation models (HESMs) are used to assist resource allocation decisions in different medical fields including diabetes [1]–[5]. These models are built on a combination of mathematical equations and computer software, and reflect key aspects of disease progression [6]. They are used to quantify the lifetime benefits and costs of alternative technologies and interventions [7].

One essential task of an HESM is to predict the risk of developing future events based on the demographic and clinical characteristics of patients, which in turn determine the expected costs and quality of life associated with different health states. To predict the risk of future events, either HESM-specific risk equations are developed [1] or equations from other studies are used [2]–[5]. For the latter case, a recent systematic review [8] showed that HESMs of diabetes usually use results from the Framingham cohort study [9], the UK Prospective Diabetes Study (UKPDS) [10] and the Diabetes Control and Complications Trial (DCCT) [11].

However, there are potential limitations in using these studies to provide data for HESMs [8]. First, the UKPDS and DCCT were randomised controlled trials, which implies that their results might not be generalisable to patients with diabetes in routine clinical practice. For example, validations of UKPDS equations in other settings have indicated that the cardiovascular risks are overestimated [12]. Second, the patients from these trials belonged to older cohorts, and might not be representative of current people with diabetes, as factors such as treatment patterns have changed since these trials were conducted. Third, the Framingham study was conducted in the USA and included only a small number of patients with diabetes (n = 337), which raises questions over the accuracy and generalisability of its results to these patients and to other settings [13]. Fourth, while some HESMs [14]–[16] have used the results of the DCCT for people with type 2 diabetes, this trial was conducted among patients with type 1 diabetes, who have different characteristics than those with type 2 diabetes. For example, the role of hyperglycaemia on the risk of cardiovascular disease (CVD) mortality might be more profound among type 1 than type 2 diabetic patients [17]. Fifth, although repeated occurrence of CVD is a well-known feature of type 2 diabetes progression [18], [19], early HESMs failed to include the risk of recurrent events [1], [14]–[16], [20], [21]; this might bias the results on costs and benefits of treatments [22]). Thus, inclusion of the risk of recurrent events in the HESM improves the accuracy and robustness of the results.

Moreover, due to the lack of data, some newer HESMs that have incorporated the risk of subsequent events have either made naive assumptions about the risk of these events (e.g. the same risk for the first and second events), or used the results of trials and studies including only a small number of participants with diabetes (e.g., the risk of recurrent myocardial infarction and recurrent stroke in the CORE Diabetes Model [4]).

To overcome these limitations, in this study we used data on history of CVD events among a large sample of people with type 2 diabetes from the Swedish National Diabetes Register (NDR), to estimate the risk of developing first and second events of four CVD events: acute myocardial infarction (AMI), heart failure (HF), non-acute ischemic heart disease (NAIHD), and stroke. We took advantage of the fact that NDR contains regular individual-level registration on outcomes and risk factors in patients with diabetes in routine clinical practice.

## Materials and Methods

### The Swedish National Diabetes Register

The Swedish National Diabetes Register (NDR) was established to enable follow-up of quality indicators and benchmarking against national guidelines, among other reasons [23]. All patients are informed (written information, oral if needed) about this quality registry. It includes individual-level demographic and clinical data on adult individuals aged ≥18 years who have provided verbal informed consent to participate (there is no requirement by Swedish law or the ethics review board that the approval to participate must be in writing). Participation in the NDR is not compulsory and patients are offered to be excluded if requested orally or in writing. Data are reported to the NDR from all hospital diabetes outpatient clinics and primary health care centres at least once a year. The study was approved by the Ethical Review Board of the University of Gothenburg.

### Explanatory variables

The variables used in the analysis were age, gender, diabetes duration, smoking, systolic and diastolic BP, HbA_1c_, total-to-HDL cholesterol ratio (TC/HDL), LDL cholesterol, history of events before diagnosis, albuminuria, and BMI (kg/m^2^). The level of HbA_1c_ was measured via high-performance liquid chromatography (HPLC) with the Mono-S method, following national standards in Sweden. All HbA_1c_ values were transformed to the National Glycohemoglobin Standardization Program (NGSP) standard levels using the formula HbA_1c_ (NGSP) = (0.923× HbA_1c_ [Mono-S]) +1.345 [24]. BP, as standard for the NDR, was given as the mean value of two readings (Korotkoff 1–5 [25]) in the supine position according to national guidelines. A smoker was defined as an individual who smoked at least one cigarette per day, or used a pipe daily, or had stopped smoking within the previous 3 months.

The proportion of missing data in the database ranged from 0.6% for HbA1c to 11% for LDL cholesterol. Table S1in File S1 shows the frequency of missing values for the variables included in the analysis. The method of last observation carried forward was applied to impute the missing values.

### Participants

In total, 29,034 individuals with type 2 diabetes in the NDR met the general inclusion criteria for the study: (1) 30–75 years old at diagnosis; (2) no missing values on explanatory variables at baseline (year 2003). To develop and validate the risk equations, the sample was randomly divided into two distinct subsamples: training (n = 21,775) and test (n = 7,259) samples. Using information in the NDR on history of CVD events, we excluded from the training sample the patients who experienced their first event after diagnosis and before 1^st^ January 2004 for first-event equations. Patients with two events after diagnosis and before 1^st^ January 2004 were excluded from the samples for the second events. The definition of type 2 diabetes was treatment with only diet or oral hypoglycaemic agent (OHA), or treatment with insulin alone or in combination with OHA and age ≥40 years at onset of diabetes.

### Follow up and definition of endpoints

For first-event equations, all patients were followed from 1^st^ January 2004 until first event or withdrawal (due to death or other reasons), or until the censoring date of 31^st^ December 2008 was reached. For second-event equations, patients were followed from the date of first event until the second event or withdrawal (due to death or other reasons), or until the censoring date. Endpoints were defined as follows:

1. AMI: non-fatal or fatal (ICD-10 code I21) or sudden death (ICD-10 codes R96.0 and R96.1).
2. HF: fatal or nonfatal (ICD-10 code I50).
3. NAIHD: fatal or nonfatal (ICD-10 codes I22, I24.8, and I24.9) including stable and unstable angina (ICD-10 codes I20.0, I20.1, I20.8, and I20.9).
4. Stroke: fatal or non-fatal (ICD-10 codes I61, I63, I64, and I67.9).

### Statistical analysis

The Prentice, Williams, and Peterson gap time model [26] was applied to estimate the hazard ratios of first and second CVD events in separate equations. Weibull proportional hazards regression was used to estimate the risk of developing these events after diagnosis of diabetes. Time since diagnosis and time since first event were used as time scales in the analysis for first and second event, respectively.

The linearity of the continuous variables was checked using design variables and residual plots [27]. The non-linear relationships were fitted using linear splines [28]. Linear continuous covariates were treated as mean-centred values in the equations (Table S2 in File S1). Except for sex, age at diagnosis, duration of diabetes at the time of first event, and history of events before diagnosis, the explanatory variables were treated as time-dependent and annual values were used in estimations. The final equation for each event was selected by backward selection processes from the full model, containing all covariates including plausible interactions. The maximum likelihood ratio test was used to test the significance of the covariates (with the 5% level used as the limit of significance).

To examine the dependency between complications, time-varying covariates showing the history of other complications were also included in the equations. These covariates were set to 0 until an event happened and 1 from that point onwards. We recorded these events if they occurred prior to the first event in question. Version 11 of the STATA software package [29] was used to estimate the equations.

### Validation

The performance of the equations was evaluated in both training and test samples. The discrimination ability of the equations was evaluated using Harrell's C statistics [30], where a value closer to one shows a better discrimination. Calibration of risk equations was assessed by a modified Hosmer-Lemeshow X^2^ test [31]. In this case, the observed and predicted numbers of events were grouped by 10 deciles of predicted risk scores. The predicted number of events for each subject was calculated using the method proposed by Gronnesby and Borgan [32]. For this, the martingale residuals for subject i were subtracted from the observed number of events for subject i.

## Results

Table 1 shows baseline characteristics of patients in both subsamples. No significant differences were found between the two samples at baseline. Tables 2 and 3 show the coefficients of the estimated first-event and second-event models. Older age at diagnosis was generally related to a higher risk of having both first and second events during the follow up. Longer duration of diabetes at the time of the first event was generally associated with higher risk of a second event.

**Table 1:** Baseline clinical and demographic characteristics of patients in training and test subsamples.

Variable		Males		Females	
		Training sample	Test sample	Training sample	Test sample
Number of patients	N	12578	4238	9197	3021
Age at diagnosis	Mean ± SD	55.36±9.28	55.33±9.24	57.15±9.55	56.89±9.66
Diabetes duration,(years)	Mean ± SD	8.92±7.14	9.02±7.08	9.02±7.17	9.15±7.37
HbA1c (%)	Mean ± SD	7.34±1.18	7.38±1.21	7.37±1.18	7.42±1.20
BMI (kg/m^2^)	Mean ± SD	28.84±4.44	28.81±4.51	29.67±5.55	29.68±5.49
Systolic BP (mmHg)	Mean ± SD	141.47±17.51	141.21±17.61	143.76±18.66	143.83±18.62
Diastolic BP (mmHg)	Mean ± SD	78.85±9.40	78.70±9.36	77.11±9.38	77.36±9.26
TC/HDL	Mean ± SD	4.23±1.29	4.20±1.29	3.95±1.25	3.96±1.27
LDL cholesterol (mmolL^−1^)	Mean ± SD	2.86±0.88	2.83±0.90	3.00±0.93	2.99±0.91
Smokers	%	15.07	15.41	14.11	14.30
Macroalbuminuria	%	7.09	6.96	4.03	3.78
Microalbuminuria	%	20.16	20.41	13.80	13.67

There were no statistically significant differences between training and test samples.

Abbreviation: BP, blood pressure; TC/HDL, total to HDL cholesterol ratio.

**Table 2:** Parameter estimates of the risk equations for first and second AMI and HF events.

		AMI		HF	
		First event	Second event	First event	Second event
N		18526	2019	19051	1646
Female		−0.2318	0.1887^a^	−0.4697	−0.1224
Age at diagnosis		0.0541	0.0254	0.0896	0.0207
HbA1c	Continuous	0.0829			0.0579
	≤7^b^			−0.2424	
	>7^b^			0.1864	
Systolic BP	Continuous	0.0079			
	≤140^b^			−0.0186	
	>140^b^			0.0050	
LDL		0.1161	0.1745		
TC/HDL		0.1712		0.1146	
BMI				0.0631	
Macroalbuminuria		0.5719	0.5478	0.7841	
Microalbuminuria		0.2176		0.6932	
Smoking		0.4938	0.2987	0.3402	0.3402
AMI history^c^		0.7469	0.7704		
HF history^c^				1.6988	
HF before first event^d^		0.6151	0.3386		
Duration at first event			0.0566		0.0268
Female * LDL			−0.2155		
Time since first event >1 year					−1.6960
Female * time since diagnosis				0.0172	
Microalbuminuria * time since diagnosis				−0.0202	
Smoking * time since first event >1 year					−0.4124
Constant		−7.8187	−2.5755	−5.3260	0.2870
P (shape parameter)		2.0537	0.7916	2.5986	0.8149

All covariates are significant at the 5% level.

Weibull proportional hazards regression with the Prentice, Williams, and Peterson gap time model was used for estimation.

Abbreviations: HF, heart failure; AMI, acute myocardial infarction; TC/HDL, total to HDL cholesterol ratio.

a . Significant at the 10% level, but significant interaction with LDL;

b . applied as splines in the equation;

c . history of event before diagnosis of type 2 diabetes;

d . HF before first AMI.

**Table 3:** Parameter estimates of the risk equations for first and second stroke and NAIHD events.

		Stroke		NAIHD	
		First event	Second event	First event	Second event
N		18992	1513	17726	1790
Female				−0.2278	0.0388^a^
Age at diagnosis		0.0727	0.0288	0.0299	
HbA1c	Continuous			0.0580	
	≤7^b^	−0.1714			
	>7^b^	0.1614			
Systolic BP		0.0063			
Diastolic BP	Continuous	0.0113			
	≤80^b^			−0.0130	
	>80^b^			0.0028	
TC/HDL		0.1121		0.1705	0.0935
BMI				0.0183	
Macroalbuminuria		0.3970		0.6346	−0.3725
Microalbuminuria		0.2551		0.1937	
Smoking		0.4010	0.8806	0.5724	
Stroke history^c^		0.9654	0.7675		
NAIHD history^c^				1.4576	
HF before first stroke		0.1826			
AMI before first stroke		0.3692			
Duration at first event			0.0518		
Female * macroalbuminuria					0.5990
Time since first event >3 years					−0.3295
Smoking * time since diagnosis				−0.0675	
Smoking * time since first event			−0.2326		
Macroalbuminuria * time since diagnosis				−0.0300	
Constant		−7.1089	−2.4119	−5.4122	−0.5534
P (shape parameter)		2.0965	0.8865	1.6704	0.4380

All covariates are significant at the 5% level.

Weibull proportional hazards regression with the Prentice, Williams, and Peterson gap time model was used for estimation.

Abbreviations: NAIHD, non-acute ischaemic heart disease; HF, heart failure; AMI, acute myocardial infarction; TC/HDL, total to HDL cholesterol ratio.

a . not significant, but significant interaction with macroalbuminuria;

b . applied as splines in the equation;

c . history of event before diagnosis of type 2 diabetes.

The shape parameter for the first event was higher than one in all equations, implying that the risk of having a first event increased with duration of diabetes. However, this parameter was less than one for second events, implying that as more time passed since the first event, the risk of experiencing a second event decreased. The results for each event are presented below.

### Risk equations

#### 1) AMI

A total of 1,084 first and 411 second AMI events were recorded based on 80,010 and 5,969 person-years, respectively. One-unit increases in HbA1c and TC/HDL were associated with 9% and 19% higher risk of having a first AMI, respectively. The hazard ratio of a 10-unit difference in systolic BP was 1.08. Patients with microalbuminuria had a 24% higher risk of a first AMI. These covariates were not independent predictors of second AMI. While the hazard ratio for a first AMI event was 0.79 (0.70–0.90) for females compared with males, it was 1.21 (0.98–1.48) for a second AMI event, all else being equal. Macroalbuminuria was associated with 77% and 73% higher risk of experiencing a first and second AMI event, respectively. LDL was a significant predictor of both AMI events, but its effect was lower for females than males for the second AMI. The risks of first and second AMI events were 64% and 35% higher for smokers than non-smokers, respectively. The risks of first and second AMI event after diagnosis were 2.11 and 2.16 times higher among patients with a history of AMI before diagnosis. Experiencing HF before the first AMI elevated the risks of first and second AMI during follow up.

#### 2) HF

During the follow up, 1,366 first and 947 second HF events were found based on 82,378 and 2,715 person-years, respectively. There were nonlinear relationships between HbA1c and systolic BP and risk of a first HF event. BMI, TC/HDL, and macroalbuminuria were associated with a higher risk of having a first HF event, but showed no independent association with a second event. The risk of a first HF after diagnosis was 5.5 times higher for patients with a history of HF before diagnosis. The risks of the first and second HF events were lower for females than males, but the difference for the first event decreased over time. The associations between microalbuminuria and the risk of first HF, and smoking and the second HF, both changed with elapsed time since diagnosis and first event. The highest rate of baseline hazard for the second event was seen during the first year after the first event.

#### 3) Stroke

During the follow up, 993 first and 314 second stroke events were observed based on 82,232 and 4,127 person-years, respectively. There was a nonlinear relationship between HbA1c and the risk of a first stroke. Ten-unit increases in systolic and diastolic BP were associated with 7% and 12% higher risk of first stroke. A one-unit increase in TC/HDL was related to a 12% higher risk of first stroke. The hazard ratios of macroalbuminuria and microalbuminuria for first stroke were 1.49 and 1.29, respectively. Patients with a history of AMI and/or HF showed higher risk of a first stroke during follow up. The associations between these covariates and risk of second stroke were not statistically significant. Smoking was associated with first and second stroke events, although the association with a second event decreased over time. Patients with a history of stroke before diagnosis had 2.6 and 2.2 times higher risk of first and second stroke after diagnosis, respectively.

#### 4) NAIHD

In total, 1,104 first and 746 second NAIHD events were recorded based on 76,174 and 4,089 person-years, respectively. A one-unit increase in HbA1c was associated with a 6% higher risk of a first NAIHD. Patients with a BMI of 30 had a 10% higher risk of a first NAIHD event after diagnosis than patients with a BMI of 25. There was a nonlinear association between diastolic BP and the risk of a first NAIHD event. TC/HDL and microalbuminuria were associated with higher risk of a first NAIHD event after diagnosis. The associations between smoking and macroalbuminuria with the risk of a first NAIHD event changed over time (implying an interaction term between smoking and time). The risk of a first NAIHD event was 4.3 times higher among patients with a history of NAIHD before diagnosis. These covariates were not independent predictors of a second NAIHD event. Sex was associated with both first and second NAIHD events, but in different directions. The effect of macroalbuminuria on the risk of a second NAIHD event differed between males and females. The baseline hazard of a second NAIHD event was higher during the first three years after the first NAIHD event.

### Validation

Table 4 shows the calibration and discrimination of the equations in training and test subsamples. Harrell's C statistics for first events ranged from 0.76 to 0.84 in the training sample and 0.75 to 0.85 in the test sample, which implies satisfactory discrimination. Calibration by means of comparing the predicted and observed number of events in ten deciles of risk score demonstrated reasonable performance in both training and test subsamples (non-significant p-values). Supplemental Figure S1 shows the observed and predicted number of first events for 10 deciles of risk scores in the test subsample. In addition, the equations for second events showed reasonable discrimination in both training (0.74–0.84) and test (0.70–0.84) subsamples. Only the equation for first HF had poor calibration (significant p-value) in the test subsample. Supplemental Figure S2 shows the observed and predicted number of second events for 10 deciles of risk scores in the test subsample.

**Table 4:** Performance of equations for the first and second events in training and test subsamples.

		Training sample		Test sample	
		C statistics (95% CI)	HL X^2^ a (P-value)	C statistics (95% CI)	HL X^2^ (P-value)
AMI	First event	0.78 (0.76–0.79)	7.30 (0.51)	0.79 (0.77–0.82)	16.33 (0.04)
	Second event	0.76 (0.74–0.79)	8.77 (0.36)	0.79 (0.74–0.84)	12.04 (0.15)
HF	First event	0.84 (0.82–0.85)	9.58 (0.30)	0.84 (0.82–0.86)	12.31 (0.14)
	Second event	0.84 (0.83–0.85)	6.11 (0.64)	0.84 (0.82–0.85)	22.67 (<0.01)
Stroke	First event	0.80 (0.78–0.82)	11.22 (0.19)	0.79 (0.76–0.82)	11.61 (0.17)
	Second event	0.74 (0.71–0.77)	8.09 (0.43)	0.70 (0.64–0.75)	9.99 (0.27)
NAIHD	First event	0.76 (0.74–0.78)	6.02 (0.65)	0.75 (0.72–0.78)	5.86 (0.66)
	Second event	0.78 (0.77–0.80)	3.76 (0.88)	0.77 (0.74–0.80)	14.07 (0.08)

Abbreviations: NAIHD, non-acute ischaemic heart disease; HF, heart failure; AMI, acute myocardial infarction.

a . Hosmer-Lemeshow X^2^ statistics.

### Example of use of the estimated risk equations

The importance of allowing different risk estimates for first and second events can be illustrated with an example. Using the risk equations for the first and second AMI, we predicted the risk of first and second AMI over 5 years for a non-smoking 58-year-old male with diabetes duration 10 years, total cholesterol 4.3 mmol/l, HDL cholesterol 1.0 mmol/l, LDL cholesterol 2.0 mmol/l, HbA1c 8.0%, systolic BP 150 mmHg, macroalbuminuria, no history of previous AMI before diagnosis, and no HF during 5 years of follow-up. To keep our example simple, the values of risk factors were assumed to remain constant during these 5 years.

With these figures, the 5-year first AMI risk was calculated as 1- exp {[-exp (−7.8187+(0.5719×1))+(0.0829×(8−7.27))+(0.0079×(150−140.92))+(0.1712×(4.3−3.89))+(0.1161×(2−2.77))+(0.0541×(58−10−56.02))]×(15 ^2.0537^−10 ^2.0537^)} = 7.32%

For a second event, we assumed that the patient had a first AMI in the 10^th^ year after diagnosis. The risk of having an AMI (second AMI event) during the 5 years following the first AMI (i.e. from 11^th^ to 15^th^ year after diagnosis) was calculated as 1− exp {[−exp (−2.5755+(0.5478×1))+(0.1745×(2−2.54))+(0.0254×(58−10−56.64))+(0.0566*(10−10))]×(5 ^0.7916^)} = 29.09%

**Figure 1:**
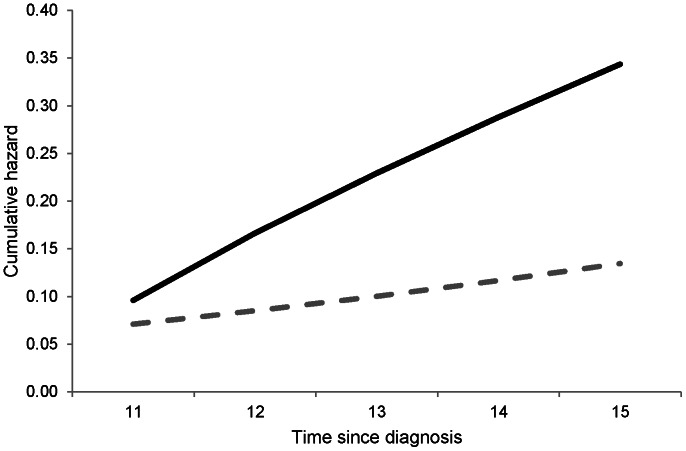
Predicted cumulative hazard of first (dashed grey line) and second (solid black line) AMI. Cumulative hazards for a non-smoking 58-year-old male with diabetes duration 10 years, total cholesterol 4.3 mmol/l, HDL cholesterol 1.0 mmol/l, LDL cholesterol 2.0 mmol/l, HbA1c 8.0%, systolic BP 150 mmHg, macroalbuminuria, no history of previous AMI before diagnosis, and no CHF during follow-up. For second AMI, it was assumed that the patient had his first AMI in the 10^th^ year after diagnosis.

In this example, 7 out of 100 patients without any event after diagnosis will experience a first AMI during the 11^th^ to 15^th^ years after diagnosis. However, 29 out of 100 patients with a first AMI in the 10^th^ year after diagnosis will experience a second AMI during the same period. Figure 1 shows the cumulative hazard of an AMI event over 5 years for these two events.

## Discussion

There is a lack of data on first and subsequent events in representative samples of patients with type 2 diabetes in routine clinical practice. As a result, the risk equations generally used in HESMs of type 2 diabetes suffer from several limitations, limiting their generalisability. To address these limitations, we estimated separate risk equations for first and second major CVD events for type 2 diabetes patients, using a large high-quality nationwide population-based database from Sweden. The results indicated heterogeneities in the effects of covariates within same CVD event (i.e., first and second events) and between different CVD events. Moreover, experiencing a first event substantially elevated the risk of a second event. The model validation indicated that the estimated equations performed well in training and test subsamples.

The risk equations in this study have several advantages compared with existing risk equations for HESMs. Firstly, our estimations reflect current routine practice and are based on a large sample (n = 29,034) from a national diabetes registry with no exclusion criteria regarding history of events before diagnosis. We estimated the event-specific equations for four major CVD events from a single database. This has two main advantages: it avoids the need to synthesise evidence from different (and sometimes heterogeneous) samples, and it captures the potential heterogeneous effect of covariates on different events. Secondly, by allowing time-varying risk factors and incorporating the history of other events, we were able to take the dependency between events into account. Moreover, allowing time-varying risk factors also accounts for potential progression of disease and other aspects including lifestyle factors that may influence the risk of CVD events. The effects of experiencing events before diagnosis on subsequent events after diagnosis were included, thus avoiding the increase in sample selection bias that comes from excluding patients with prior events. In addition, the randomly selected test sample in the validation analysis had a large number of patients (n = 7259), which strengthens the analysis.

We found that while the risks of first events increased with increasing time since diagnosis, the risks of second events decreased with increasing time since first event. One explanation for this is that patients who have experienced a first event will get more treatment and care [33]. In addition, this implies that the initial time after a first event is the most hazardous period for patients to have a subsequent event; this aspect should be incorporated into the HESM. The associations between the risk factors and first CVD events were in the same direction as those found in previous studies [34]–[36], which implies that patients with type 2 diabetes share many similar features. Moreover, we found that gender, age at diagnosis, smoking, and duration of diabetes at the time of the first events were the most important predictors of second events. The results indicated that CVD events are not independent, and experiencing one event increases the risk of having others. This highlights the importance of taking into account the dependency between events, as recommended by the American Diabetes Association [6].

An updated analysis [37] of UKPDS data showed that the effects of covariates are not constant between the first and second events, which is in line with our findings. This illustrates how the accuracy of an HESM could be increased by taking the heterogeneity between the risk of first and subsequent events into account. Failure to account for heterogeneity might lead to an underestimation of the costs and an overestimation of patients' utilities. Moreover, if an intervention is effective for preventing the occurrence of recurrent events, an economic analysis including only the first event might lead to biased results.

To demonstrate how the results of the current study might lead to more accurate conclusions, we considered two examples. Although females were less susceptible to a first AMI event, they were more vulnerable for a second AMI event. Thus, if only the first event is taken into consideration, treating females would appear to be less cost-effective; however, if the higher risk of second events is then considered, the previous conclusion might be incorrect. Another example is that while BMI was not associated with the risk of AMI and stroke events, it was an independent predictor of first HF and NAIHD. This shows how specific-event equations may lead to more accurate results than estimating one equation for all events pooled as “CVD events” [38]–[40].

The results of the current study should be interpreted in light of some limitations. As we used register-based data, the possibility of error in the recording of data including ICD-10 codes may be a source of information bias. Moreover, the non-compulsory nature of participation in the NDR is a potential source of selection bias if, for example, older or sicker patients are less willing to participate. However, it is estimated that the NDR currently covers more than 90% of all patients in hospital outpatient clinics and almost 80% of all patients in primary care in Sweden [41], and so selection bias is not likely to be a significant problem in our analysis.

To summarise, the current study provides separate risk equations for the first and second events to improve the accuracy and robustness of HESMs. Moreover, these equations are crucial steps in developing HESMs for type 2 diabetes in Sweden. Although these equations performed well in the test subsample, future research should validate them in other populations, in order to evaluate the feasibility of transferring them to other settings.

## Supporting Information

File S1 **This file contains Tables S1 and S2. Table S1. Frequency distribution of missing values. Table S2. The values for mean-centering of continuous covariates in the risk equations.**
(DOC)

Figure S1 **Predicted (black bar) and observed (grey bar) number of first events in the test sample.**
(TIF)

Figure S2 **Predicted (black bar) and observed (grey bar) number of second events in the test sample**.
(TIF)
