# Supplementary material for: Towards Renewed Health Economic Simulation of Type 2 Diabetes: Risk Equations for First and Second Cardiovascular Events from Swedish Register Data
Source: PLoS One. 2013 May 9;8(5):e62650. doi: 10.1371/journal.pone.0062650 (PMC3650043; doi:10.1371/journal.pone.0062650)
Supplement: File S1 — This file contains Tables S1 and S2. Table S1. Frequency distribution of missing values. Table S2. The values for mean-centering of continuous covariates in the risk equations. (DOC) [file pone.0062650.s001.doc]

Table S1- Frequency distribution of missing values

| **Variable** | **No. of missing values (%)** |
| --- | --- |
| BMI | 4828 (2.4) |
| HbA1c | 1132 (0.6) |
| Systolic BP | 2004 (1.0) |
| Diastolic BP | 2004 (1.0) |
| TC: HDL | 20658 (10.1) |
| LDL | 22597 (11.0) |
| Smoking | 9482 (4.6) |
| Macroalbuminuria | 14738 (7.2) |
| Microalbuminuria | 22259 (10.9) |

Table S2. The values for mean-centering of continuous covariates in the risk equations.

| **Variable** | **AMI** | | **HF** | | **Stroke** | | **NAIHD** | |
| --- | --- | --- | --- | --- | --- | --- | --- | --- |
|  | **First event** | **Second event** | **First event** | **Second event** | **First event** | **Second event** | **First event** | **Second event** |
| Age at diagnosis | 56.02 | 56.64 | 56.01 | 58.74 | 56.00 | 57.63 | 56.02 | NS |
| HbA1c (%) | 7.27 | NS | S | 7.47 | S | NS | 7.26 | NS |
| Systolic BP | 140.92 | NS | S | NS | 140.70 | NS | NS | NS |
| Diastolic BP | NS | NS | NS | NS | 76.80 | NS | S | NS |
| LDL | 2.77 | 2.54 | 2.74 | NS | NS | NS | NS | NS |
| TC/HDL | 3.89 | NS | 3.90 | NS | 3.89 | NS | 3.89 | 3.97 |
| BMI | NS | NS | 29.17 | NS | NS | NS | 29.17 | NS |
| Duration at time of first event | NA | 10.00 | NA | 12.27 | NA | 10.71 | NA | 9.89 |

NS: non-significant, NA: not-applied in the model, S: used as splines in the model.
