# Supplementary figures and images for: Towards Renewed Health Economic Simulation of Type 2 Diabetes: Risk Equations for First and Second Cardiovascular Events from Swedish Register Data
Source: PLoS One. 2013 May 9;8(5):e62650. doi: 10.1371/journal.pone.0062650 (PMC3650043; doi:10.1371/journal.pone.0062650)

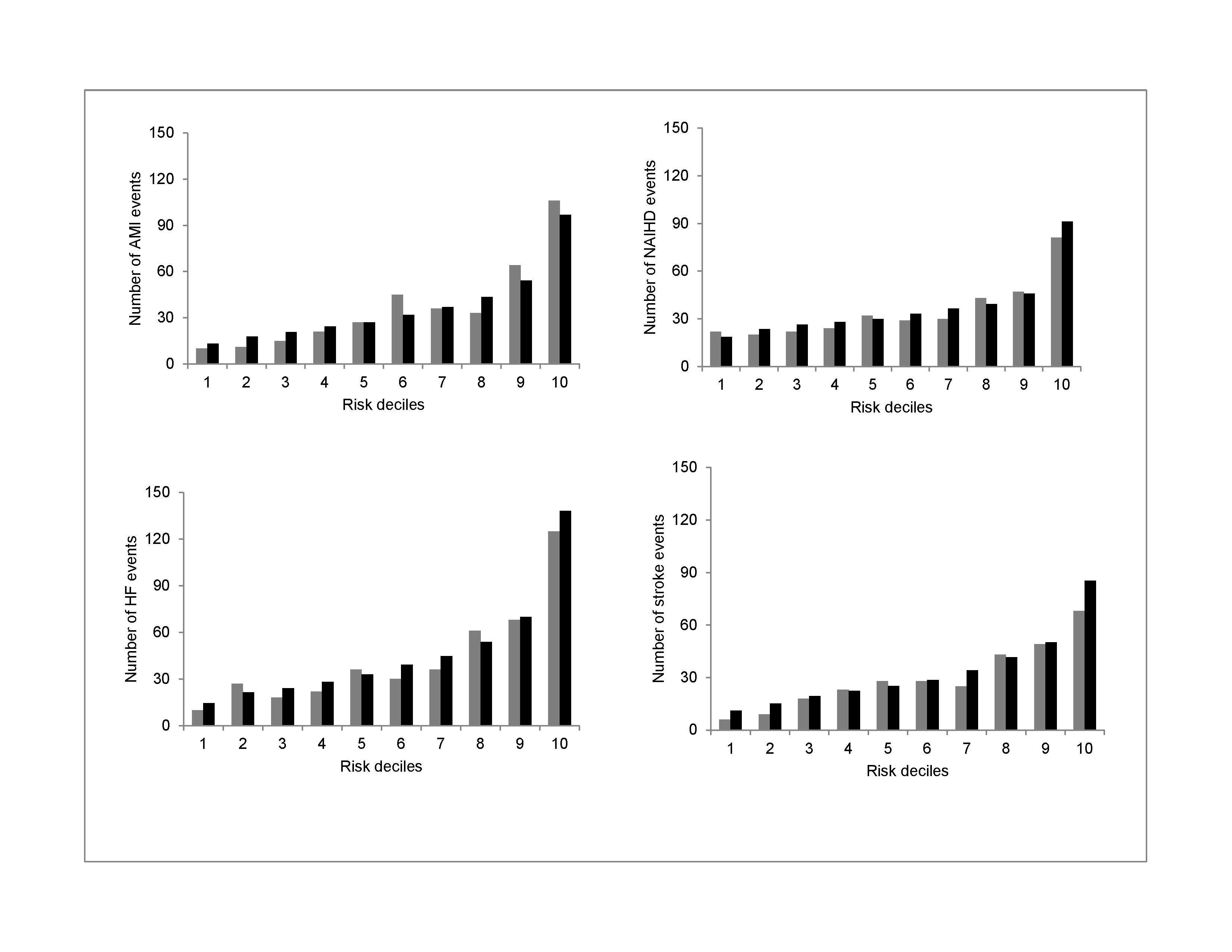

Supplement: Figure S1 — Predicted (black bar) and observed (grey bar) number of first events in the test sample. (TIF) [file pone.0062650.s002.tif]

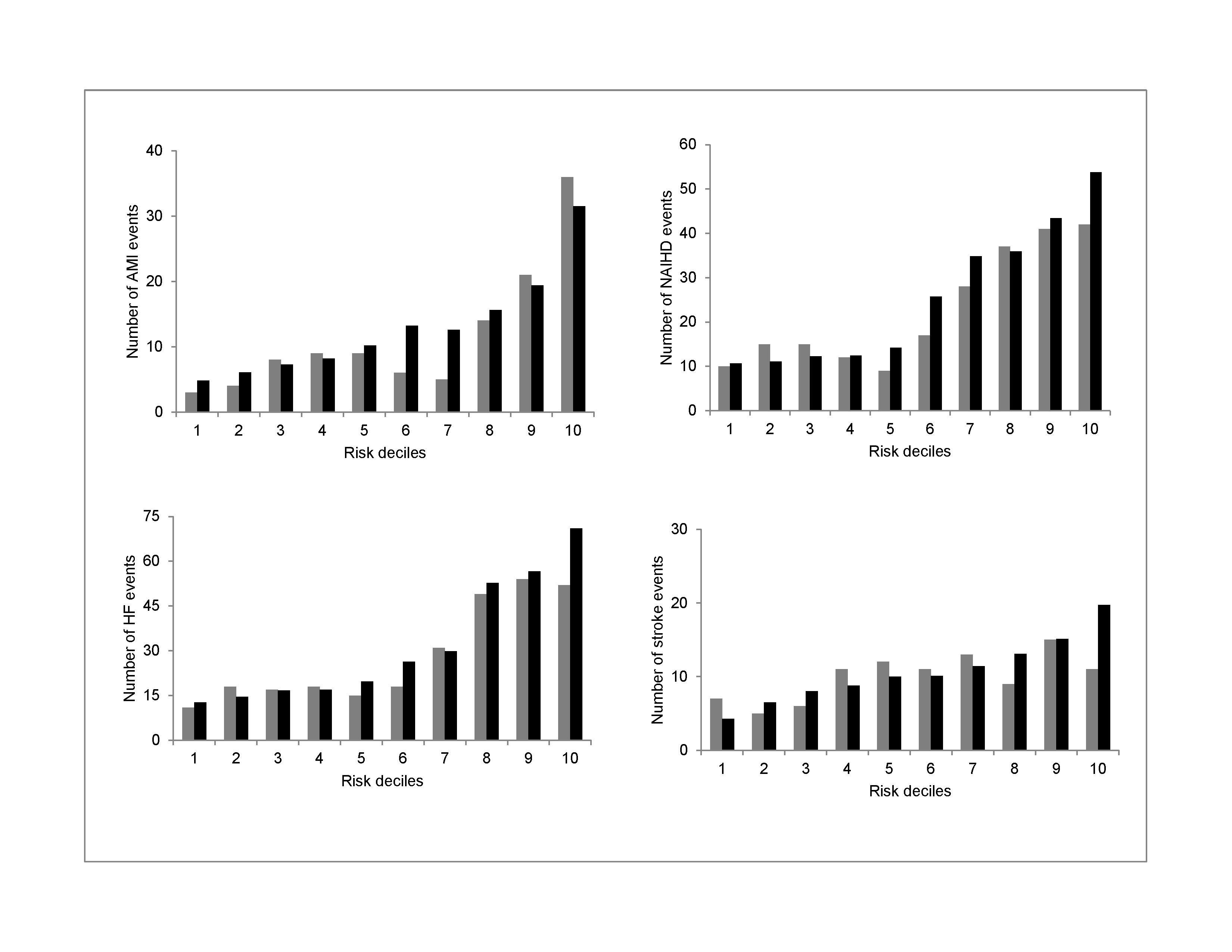

Supplement: Figure S2 — Predicted (black bar) and observed (grey bar) number of second events in the test sample. (TIF) [file pone.0062650.s003.tif]
